# Relationship of hyperemesis gravidarum with platelet crit, hemoglobin-to-red cell distribution width ratio, and neutrophil-to-lymphocyte ratio

**DOI:** 10.1590/1806-9282.20241754

**Published:** 2025-07-07

**Authors:** Kazım Uçkan, Yusuf Başkiran, İzzet Çeleğen

**Affiliations:** 1 Van Yuzuncu Yil University, Faculty of Medicine, Gynecology and Obstetrics Clinic – Van, Turkey.; 2 Van Yuzuncu Yil University, Faculty of Medicine, Departtment of Public Health – Van, Turkey.

**Keywords:** Hyperemesis gravidarum, Inflammation, Complete blood count, Pregnancy complications, Biomarkers

## Abstract

**OBJECTIVE:**

The aim of this study was to investigate the relationship between the severity of hyperemesis gravidarum disease and subclinical inflammatory factors such as platelet crit, hemoglobin-to-red cell distribution width ratio, neutrophil-to-lymphocyte ratio, which are known to be closely associated with inflammation in patients with hyperemesis gravidarum.

**METHODS:**

This retrospective case–control study was conducted between December 2020 and December 2021. A total of 215 pregnant women, 102 with hyperemesis gravidarum and 113 healthy pregnant women, were included in the study. Hyperemesis gravidarum patients were divided into three groups according to the modified Pregnancy-Unique Quantification of Emesis and nausea classification as mild (n=38), moderate (n=32), and severe (n=32).

**RESULTS:**

Platelet crit, hemoglobin-to-red cell distribution width ratio, and neutrophil-to-lymphocyte ratio values were found to be statistically significantly higher in the hyperemesis gravidarum group compared to the control group (p<0.05). There was a mild-to-severe increase in platelet crit, hemoglobin-to-red cell distribution width ratio, and neutrophil-to-lymphocyte ratio values in hyperemesis gravidarum patients (p<0.05). Logistic regression analysis revealed that a one-unit increase in platelet crit, hemoglobin-to-red cell distribution width ratio, and neutrophil-to-lymphocyte ratio resulted in a 2.14-, 1.41-, and 2.36-fold increase in hyperemesis gravidarum risk, respectively.

**CONCLUSIONS:**

Platelet crit, hemoglobin-to-red cell distribution width ratio, and neutrophil-to-lymphocyte ratio are inflammatory markers that increase in patients with hyperemesis gravidarum and have predictive value for the development of hyperemesis gravidarum. In our study, we suggested the use of a new prognostic marker for patients with hyperemesis gravidarum. We believe that our study will be a source for future studies on hyperemesis gravidarum.

## INTRODUCTION

Hyperemesis gravidarum (HEG), characterized by severe nausea and vomiting during pregnancy, affects approximately 0.3–2% of pregnancies and is associated with both maternal and fetal morbidity^1^. The pathophysiology of HEG remains unclear, but proposed mechanisms include hormonal changes, gastrointestinal dysmotility, genetic predisposition, and systemic inflammation^2,3^. The lack of objective and widely accepted diagnostic criteria continues to complicate clinical management.

Recent studies have highlighted the role of systemic inflammation in various chronic and acute diseases and have proposed that hemogram-derived indices may serve as accessible inflammatory markers^4^. In particular, neutrophil-to-lymphocyte ratio (NLR), platelet crit (PCT), and the relatively novel hemoglobin-to-red cell distribution width ratio (HRR) have gained attention for their predictive value in systemic inflammation, autoimmune disease, and even infection^5–7^. Inflammatory indices such as NLR and PCT have been found to be significantly elevated in COVID-19^8^, autoimmune thyroiditis^9^, and various thyroid disorders^10^, supporting their potential utility in HEG, a condition with an increasingly recognized inflammatory component.

Aktas et al. have shown that both NLR and PCT are elevated in diverse clinical settings and may reflect subtle inflammatory shifts not captured by traditional acute-phase reactants^8–10^. Furthermore, hemogram-based inflammatory indices are cost-effective, reproducible, and rapidly obtainable—making them highly valuable for use in obstetric settings, especially in under-resourced environments^7^.

Despite the expanding use of these markers, the relationship between HRR and HEG has not yet been explored in the literature. Given the strong predictive capacity of HRR in cardiovascular and oncologic contexts^11,12^, it is logical to investigate its role in pregnancy-related inflammatory states such as HEG. This study aimed to evaluate whether PCT, HRR, and NLR are significantly elevated in patients with HEG and whether these markers can be used to predict disease severity.

## METHODS

### Study design

This retrospective case–control study was conducted in the Gynecology and Obstetrics Unit of Van Training and Research Hospital. As a retrospective analysis, no prospective randomization or control over confounding variables (such as nutritional status or hormonal levels) was possible.

### Participants

This retrospective case–control study was conducted in the Gynecology and Obstetrics Unit of Van Training and Research Hospital. The study examined the records of pregnant women hospitalized with a diagnosis of HEG between December 2020 and 2021. A total of 213 pregnant women were included in the study: 102 women diagnosed with HEG and 113 healthy pregnant women between 5 and 16 weeks of gestation. Due to the study's design and sample availability, subgroup sizes were relatively limited, particularly when stratified by disease severity. The inclusion criteria for HEG diagnosis were defined as a weight loss of 5% or more from the beginning of pregnancy, vomiting at least three times a day, and a ketonuria value of +1 or higher in a urinalysis test.

### Ethical approval and consent

Ethics approval was obtained from the Van Ministry of Health University Training and Research Hospital Clinical Research and Ethics Committee (decision no: 2022/02-01, dated 18.01.2022). Verbal informed consent was provided by all participants.

### Data source

Data were retrieved from medical records, including Pregnancy-Unique Quantification of Emesis and nausea (PUQE) scores, body mass index (BMI), gestational age (based on the last menstrual period and sonography), and hematological parameters (HRR, eosinophil-to-lymphocyte ratio [ELR], NLR, platelet-to-lymphocyte ratio [PLR], platelet distribution width [PDW], mean platelet volume [MPV], PCT, white blood cell [WBC] count, hemoglobin [Hb], platelet count [PLT], and ketonuria results).

### Exclusion criteria

Participants were excluded if they smoked or had urinary tract infections, psychological or gastrointestinal disorders, thyroid or inflammatory diseases, eating disorders, multiple pregnancies, or pregnancies via assisted reproductive techniques.

### Data collection and assessment

Hematological ratios and related parameters were collected from medical records to evaluate their association with HEG.

### Statistical analysis

Statistical analysis was performed using SPSS version 22.0. Shapiro-Wilk test was used to assess whether the variables followed normal distribution or not. Variables were reported as mean (minimum:maximum) values. A Mann-Whitney U test was used to compare patients in the HEG and control groups. Receiver operating characteristic (ROC) curve analysis was used to identify the optimal cutoff values of PCT, HRR, and NLR for diagnosing severe HEG with maximum sensitivity and specificity. A Kruskal-Wallis test was performed to compare patients with mild, moderate, and severe HEG. Moreover, a Mann-Whitney U test was used for pairwise comparison. To identify the independent risk factors affecting the development of HEG, binary logistic regression analysis was performed with the backward selection procedure. No specific measures were taken to control for potential confounders such as pre-existing nutritional deficiencies, gastrointestinal comorbidities, or hormonal variations. This limitation stems from the retrospective nature of the study and the reliance on pre-recorded hospital data. The level of significance was set at α=0.05.

## RESULTS

The demographic characteristics and laboratory findings of the patients are shown in Table 1. There was no significant difference between the HEG and control groups in terms of age, gestational age, parity, BMI, and thyroid functions (p>0.05). However, WBC, Hb, PCT, HRR, and NLR values were higher in the HEG group (p<0.05).

**Table 1:** Comparison of demographic and laboratory parameters between the groups.

	Control group (n=113)		HEG group (n=102)		p
Age	29.49	(18.00–42.00)	29.89	(18–42)	0.63^a^
Gravida	1	(1–4)	1	(1–4)	0.67^a^
Gestational age (week)	10.53	(7–12)	10.34	(7.00–12)	0.43^a^
Parity (number)	1	(0:5)	1	(0:5)	0.30^a^
Body mass index (kg/m^2^)	26.70	(22.10–30.10)	26.30	(22.10–30.20)	0.17^a^
TSH (U/L)	0.32	0.28–3.6	0.31	0.29–3.4	0.23^a^
fT4 (pmol/L)	13.43	13–18	13.39	12–19	0.34^a^
WBC	6.53	(5.30–8.20)	9.79	(4.73–15.80)	**0.001** a
Hb (g/dL)	12.93	±1.06	13.47	±1.19	**0.001** b
MCV	92.22	(90.50–96.00)	92.30	(83.10–99.70)	0.64^a^
PLT (10^3^/μL)	265.63	(195–355)	271.48	(166–409)	0.48^a^
PCT (%)	0.23	(0.16–0.23)	0.31	(0.17–0.58)	**0.001** a
RDW (%)	16.49	(16.10–16.50)	16.47	(11.7–16.90)	0.43^a^
MPV (fL)	9.20	±0.42	9.17	±0.95	0.23^b^
HRR (%)	0.98	(0.79–1.17)	1.0063	(0.66–1.27)	**0.04** a
PLR (%)	182.19	(62.10–581.97)	187.04	(54.43–670.50)	0.07^a^
NLR (%)	2.88	(0.42–8.07)	5.43	(1.15–21.8)	**0.001** a
MLR (%)	0.26	(0.06–0.56)	0.27	(0.12–1.03)	0.15^a^
ELR (%)	0.05	(0.001–0.32)	0.04	(0.01–0.29)	0.09^a^

The levels of categories are presented as the mean±standard deviation for parametric variables and median (min–max) for nonparametric variables. Statistically significant values are denoted in bold. TSH: thyroid-stimulating hormone; fT4: free T4; WBC: white blood cell; Hb: hemoglobin; MCV: mean corpuscular volume; PLT: platelet count; PCT: platelet crit; RDW: red cell distribution width; MPV: mean platelet volume; HRR: hemoglobin-to-red cell distribution width ratio; PLR: platelet-to-lymphocyte ratio; NLR: neutrophil-to-lymphocyte ratio; MLR: monocyte-to-lymphocyte ratio; ELR: eosinophil-to-lymphocyte ratio; HEG: hyperemesis gravidarum.

a Mann-Whitney U test, and

b independent-sample t-test.

In the HEG subgroups (mild, moderate, and severe), based on laboratory values, significant differences were observed in PCT, HRR, and NLR levels. Specifically, PCT, HRR, and NLR values were significantly higher in the severe group compared to the mild and moderate groups, while there was no significant difference between the mild and moderate groups. This trend indicated that PCT, HRR, and NLR values increased progressively from mild-to-severe HEG cases.

The diagnostic utility of PCT, HRR, and NLR for severe HEG was evaluated using ROC analysis, revealing that the areas under the curve (AUC) for PCT, HRR, and NLR were 0.80, 0.76, and 0.71, respectively. Threshold values associated with an increased risk of severe HEG were identified as PCT>0.29, HRR>1.03, and NLR>4.93, all with p<0.05. PCT demonstrated the highest sensitivity (78%) and specificity (72%), followed by HRR with a sensitivity of 75% and a specificity of 74%, and NLR with a sensitivity of 71% and a specificity of 60% (Table 2).

**Table 2:** Receiver operating characteristic analysis results for inflammatory variables.

Variables	AUC	SE	p	Predictive value	Sensitivity	Specificity	95%CI	
PCT	0.80	0.47	**0.001**	0.29	0.78	0.72	0.71	0.89
HRR	0.76	0.51	**0.001**	1.03	0.75	0.74	0.67	0.86
NLR	0.71	0.57	**0.001**	4.93	0.71	0.60	0.60	0.82

AUC: area under the curve; SE: standard error; CI: confidence interval; PCT: platelet crit; HRR: hemoglobin-to-red cell distribution width ratio; NLR: neutrophil-to-lymphocyte ratio. Statistically significant values are denoted in bold.

Logistic regression analysis showed that a one-unit increase in PCT, HRR, and NLR was associated with a 2.14-, 1.41-, and 2.36-fold increase in the risk of developing HEG, respectively (Table 3).

**Table 3:** The effects of platelet crit, hemoglobin-to-red cell distribution width ratio, and neutrophil-to-lymphocyte ratio on hyperemesis gravidarum development by binary logistic regression analysis.

Variables	B	OR	95%CI	P
PCT	0.76	2.14	1.65–2.79	**0.001**
HRR	0.34	1.41	1.12–1.76	**0.03**
NLR	0.863	2.36	1.66–3.36	**0.001**

OR: odds ratio; CI: confidence interval; PCT platelet crit; HRR: hemoglobin-to-red cell distribution width ratio; NLR: neutrophil-to-lymphocyte ratio. Statistically significant values are denoted in bold.

## DISCUSSION

HEG is a condition that causes severe nausea and vomiting in early pregnancy and often requires hospitalization. HEG has a pathophysiological mechanism due to many causes^13^. HEG may be severe enough to require hospitalization. It may even progress to central pontine myelinolysis and Wernicke's encephalopathy. Therefore, early diagnosis and treatment of HEG are essential for maternal and child health^14^.

Although the exact mechanism underlying HEG is not fully understood, inflammation has been suggested as a contributing factor. Hormonal changes, particularly rising levels of estrogen and human chorionic gonadotropin (hCG), are believed to trigger a cascade of immune responses. These responses include increased production of cytokines such as interleukin-6 (IL-6), tumor necrosis factor-alpha (TNF-α), and other proinflammatory mediators, which may exacerbate nausea and vomiting symptoms^15,16^.

Although the link between HEG and inflammation is not fully understood, studies on markers of inflammation in HEG patients suggest a strong association between them^17,18^.

The rationale for studying inflammatory biomarkers in HEG is based on their ability to reflect underlying systemic inflammation, which may correlate with disease severity. These markers—including procalcitonin (PCT), NLR, and HRR—can be easily obtained from routine blood tests and offer a practical approach to risk stratification in pregnant women. Demir Cendek et al. demonstrated that blood-based inflammatory and nutritional parameters are significantly altered in women with HEG, supporting their potential diagnostic and prognostic utility^19^. Similarly, elevated ischemia-modified albumin (IMA) levels and decreased total-sulfhydryl concentrations observed in HEG patients suggest that subclinical inflammation and oxidative stress are both implicated in the pathophysiology of the condition^20^.

PCT, an inflammatory marker obtained from a complete blood count, has been reported to have prognostic and predictive properties in various diseases such as gynecological and gastrointestinal malignancies, autoimmune diseases, and coronary artery diseases^21,22^. There are not many studies on the relationship between PCT and HEG. In the study by Tayfur et al., PCT values were found to be higher in women with HEG. In the same study, mild, moderate, and severe HEG cases were compared, and it was reported that PCT values were higher in severe HEG cases^23^. In our study, PCT values in the HEG group were found to be significantly higher than in the control group. In addition, mild, moderate, and severe HEG cases were compared, and PCT values increased as one went from the mild group to the severe group. According to the ROC analysis result, the rate of PCT>0.29 was determined statistically, and this parameter was significantly associated with an increased risk of severe HEG disease. Logistic regression analysis revealed that a one-unit increase in PCT resulted in a 2.14-fold increase in HEG risk.

HRR is a recently used inflammatory marker derived from Hb and red cell distribution width (RDW), which are complete blood count parameters used in routine practice. In addition, it has been shown to be a bad prognostic factor alone in many cancers, such as stomach cancer and lung cancer^24,25^. Çintesun et al. found no significant difference in RDW between the HEG and control groups in their study^21^. Similarly, in our study, no significant difference was observed between the control and HEG groups in terms of RDW. There is no study in the literature about HRR in patients with HEG. We believe that the data we obtained in this study will lay the groundwork for future studies. In our study, HRR levels were found to be significantly higher in the HEG group than in the control group. When HEG subgroups were examined, HRR values increased significantly from mild to severe. According to the ROC analysis result, the rate of HRR>1.03 was determined statistically, and this parameter was significantly associated with an increased risk of severe HEG disease. Logistic regression analysis revealed that a one-unit increase in HRR resulted in a 1.41-fold increase in HEG risk.

NLR has been reported to increase in gastrointestinal and gynecological malignancies, heart diseases, and various inflammatory conditions^26,27^. Looking at the literature, there are few studies on the severity of NLR and HEG. Soysal et al. reported that NLR levels were higher in the patient group. In the same study, a significant correlation was found between increased levels of ketonuria and NLR^28^. In another study, NLR levels were found to be high in HEG patients. However, no correlation was found between NLR values and the degree of ketonuria^21^. In the study by Kan et al., NLR levels were higher in the HEG group. However, no correlation was found between the severity of the disease and NLR values^29^. In another similar study, a significant correlation was found between NLR levels and HEG groups^30^. In our study, NLR values were higher in the HEG group. NLR values increase as one moves from the mild group to the severe group. According to the ROC analysis result, the rate of NLR>4.93 was determined statistically, and this parameter was significantly associated with an increased risk of severe HEG disease. Logistic regression analysis revealed that a one-unit increase in NLR resulted in a 2.36-fold increase in HEG risk.

Another important consideration is the role of potential confounding factors such as hormonal fluctuations and nutritional deficiencies. Elevated hCG and estrogen levels can directly contribute to nausea and vomiting, independent of inflammation. Additionally, thiamine, folate, and iron deficiencies—which are common in pregnant women—may worsen HEG symptoms and indirectly influence inflammatory markers. Kaplan et al. and Kuscu et al. both highlighted the importance of evaluating cytokine levels and their correlation with clinical symptoms in the context of these confounders^15,16^. Future studies should take such variables into account to better clarify the causal relationships between inflammation and HEG.

Limitations of this study: First, the patient data were obtained from a single center, the number of patients was not large, and the study design was retrospective. Therefore, it may be subject to selection bias and lacks the ability to control for possible confounding variables such as nutritional deficiencies, hormonal status, and underlying systemic conditions. Moreover, the single-center nature of the study may limit the generalizability of our findings to broader populations or different clinical settings. Second, only HRR, PLR, NLR, ELR, monocyte-to-lymphocyte ratio (MLR), and other hematological parameters were used as inflammatory markers, and biochemical inflammatory markers such as C-reactive protein (CRP) or ILs were not evaluated. Furthermore, the relatively limited sample size may have reduced the statistical power of subgroup comparisons and may not have fully captured population-level variations.

The strength of our study is that it is the first to investigate the association of HRR with HEG, providing a basis for future prospective, multi-center studies.

## CONCLUSION

PCT, HRR, and NLR are inflammatory markers that increase in patients with HEG and have a predictive value for the development of HEG. In our study, we suggested using the Hb/RDW ratio as a new prognostic marker for patients with HEG. HRR, PCT, and NLR can be implemented at no additional cost. Since the relationship of HRR with HEG has not been definitively investigated, we cannot make a definitive statement about its clinical use yet. As more data on HRR levels are collected, HRR may be a marker of HEG. We believe that our study will be a source for future studies on the subject.

## Data Availability

The datasets generated and/or analyzed during the current study are available from the corresponding author upon reasonable request.
